# A Systematic Review of Platelet‐Rich Plasma Versus Platelet‐Rich Fibrin for Periorbital Rejuvenation

**DOI:** 10.1111/jocd.70524

**Published:** 2025-11-05

**Authors:** Catherine F. Sollitto, Maxim Narduzzi, Claire Wolinsky

**Affiliations:** ^1^ New York Institute of Technology College of Osteopathic Medicine Old Westbury New York USA; ^2^ Department of Dermatology The Mount Sinai Hospital New York New York USA

**Keywords:** cosmetic dermatology, facial rejuvenation, infraorbital hollows, periorbital hyperpigmentation, platelet analogues, platelet‐rich fibrin, platelet‐rich plasma

## Abstract

**Background:**

Platelet‐rich plasma (PRP) and platelet‐rich fibrin (PRF) have become popular autologous options for facial rejuvenation due to their regenerative potential and favorable safety profiles. The periorbital region remains one of the most challenging treatment sites, as conventional approaches such as hyaluronic acid fillers carry a risk of complications and may not provide natural, sustained results.

**Aim:**

This systematic review aims to compare injectable PRP and PRF in periorbital rejuvenation, with emphasis on treatment protocols, efficacy, adverse effects, and patient‐reported outcomes.

**Methods:**

A PRISMA‐guided search was conducted using PubMed, Embase, and Wiley Library. Included studies were evaluated for differences in preparation, injection techniques, frequency of treatment, clinical outcomes, adverse events, and satisfaction.

**Results:**

A total of 14 studies met inclusion criteria. Across studies, PRF was associated with improvements in skin texture, wrinkles, and crepiness, while PRP showed stronger evidence for treating hyperpigmentation. Both modalities demonstrated favorable safety profiles and high patient satisfaction, with only mild, transient adverse effects reported. Longevity of results remains unclear: PRF improvements often diminished by 6 months, whereas PRP outcomes for pigmentation were sustained at similar intervals.

**Conclusion:**

PRF shows promise in improving periorbital texture and fine lines, while PRP appears more effective for pigmentation, but current evidence does not support the superiority of one modality over the other. However, the long‐term durability of PRF remains uncertain, with improvements often diminishing by 6 months. Larger randomized controlled trials with standardized, objective outcome measures are needed to clarify their long‐term efficacy.

## 1 Introduction

The periorbital region is a notoriously challenging region to treat for aesthetic purposes and yet one of the most frequently requested due to its proclivity to show signs of aging. Patient concerns often include infraorbital hollows, hyperpigmentation, and wrinkles, all of which may be treated with distinct techniques that vary in efficacy and patient satisfaction. Currently, the most common in‐office treatment option is hyaluronic acid (HA) fillers due to their safety and efficacy in filling infraorbital hollows and smoothing wrinkles [1, 2]. However, HA fillers also carry risks, including vascular occlusion, visual compromise, nodules, asymmetry, and the Tyndall effect [3]. Thus, more patients have been opting to dissolve their filler in recent years and shift their preference toward more natural alternatives with fewer complications [4, 5].

Platelet‐rich plasma (PRP) and platelet‐rich fibrin (PRF) have gained traction over the last decade as minimally invasive options for facial rejuvenation [6, 7]. Studies describing the regenerative and healing properties of PRP in a clinical setting have existed since the late 1980s [8]. Since then, it has been studied as a promising tool for natural tissue repair and regeneration in a variety of fields, including cosmetic dermatology. PRP is often utilized to improve skin texture, tone, density, and pigmentation, as well as for its ability to treat alopecia and mitigate acne scars. Following its more recent development, PRF has been used to treat similar concerns with the goal of enhanced results. Both modalities are generally injected in facial regions prone to volume deficit, such as the nasolabial folds, malar areas, and tear troughs, in an effort to restore volume loss via collagen production, and can be used in conjunction with HA filler to augment its effect [9, 10, 11, 12].

Preparation of PRP occurs via a double‐spin centrifugation process using the patient's whole blood to obtain an increased concentration of autologous platelets suspended in plasma. The platelets contain growth factors such as platelet‐derived growth factor (PDGF), transforming growth factor β (TGF‐β), vascular endothelial growth factor (VEGF), epidermal growth factor (EGF), insulin‐like growth factor (IGF), and fibroblast growth factor (FGF). All of these factors aid in neovascularization and wound healing via recruitment, activation, and differentiation of stem cells. PDGF, specifically, promotes proliferation of fibroblasts and smooth muscle cells which contributes to collagen synthesis [13, 14]. EGF also holds a crucial role in skin regeneration as it stimulates pro‐inflammatory cytokine secretion and epithelialization, thus expediting the healing process [15].

However, PRP must be combined with an external anticoagulant and its release of growth factors is rapid and transient. Thus, PRF was developed to address such limitations. PRF undergoes a shorter, slower, single‐spin centrifugation process that creates a three‐dimensional fibrin matrix. This matrix acts as a structural scaffold for tissue repair and cellular migration, as it entraps platelets and leukocytes during centrifugation. This results in prolonged stem cell stimulation, greater release of growth factors for up to 2 weeks, and enhanced healing capabilities [13, 16, 17, 18].

Although both PRP and PRF offer favorable adverse event profiles and the convenience of immediate availability, the current evidence supporting their efficacy in aesthetic dermatology is mixed [6]. Not only is aesthetic improvement relatively subjective, but the body's ability to produce collagen from growth factors is neither guaranteed nor uniform among patients. Moreover, PRF has been introduced into clinical practice much more recently and lacks research in comparison to PRP. This necessitates a thorough investigation into the literature and comparison of both modalities to better understand the perceived aesthetic benefits, efficacy, and overall patient satisfaction yielded by each procedure. Specifically, this review aims to evaluate the efficacy of injectable PRP vs. PRF in treating periorbital hyperpigmentation (POH), wrinkles, texture, and hollowing, as well as discover which protocols yield the best results and highlight areas for future investigation.

## 2 Materials and Methods

### 2.1 Search Strategy

A systematic literature search was conducted across multiple databases until September 1, 2025 to explore the aesthetic outcomes and patient satisfaction of injectable PRP and PRF in the periorbital region. PubMed, MEDLINE, and Wiley Library were searched using the following terms: (platelet‐rich plasma OR platelet rich plasma OR PRP OR platelet‐rich fibrin OR platelet rich fibrin OR PRF) AND (discoloration OR hyperpigmentation OR dark circles OR collagen production OR aesthetic benefit OR hollows OR skin texture) AND (periorbital OR periocular OR under eyes OR infraorbital). The Preferred Reporting Items for Systematic Reviews and Meta‐Analyses (PRISMA) 2020 guidelines were followed accordingly.

### 2.2 Eligibility Criteria

**FIGURE 1:**
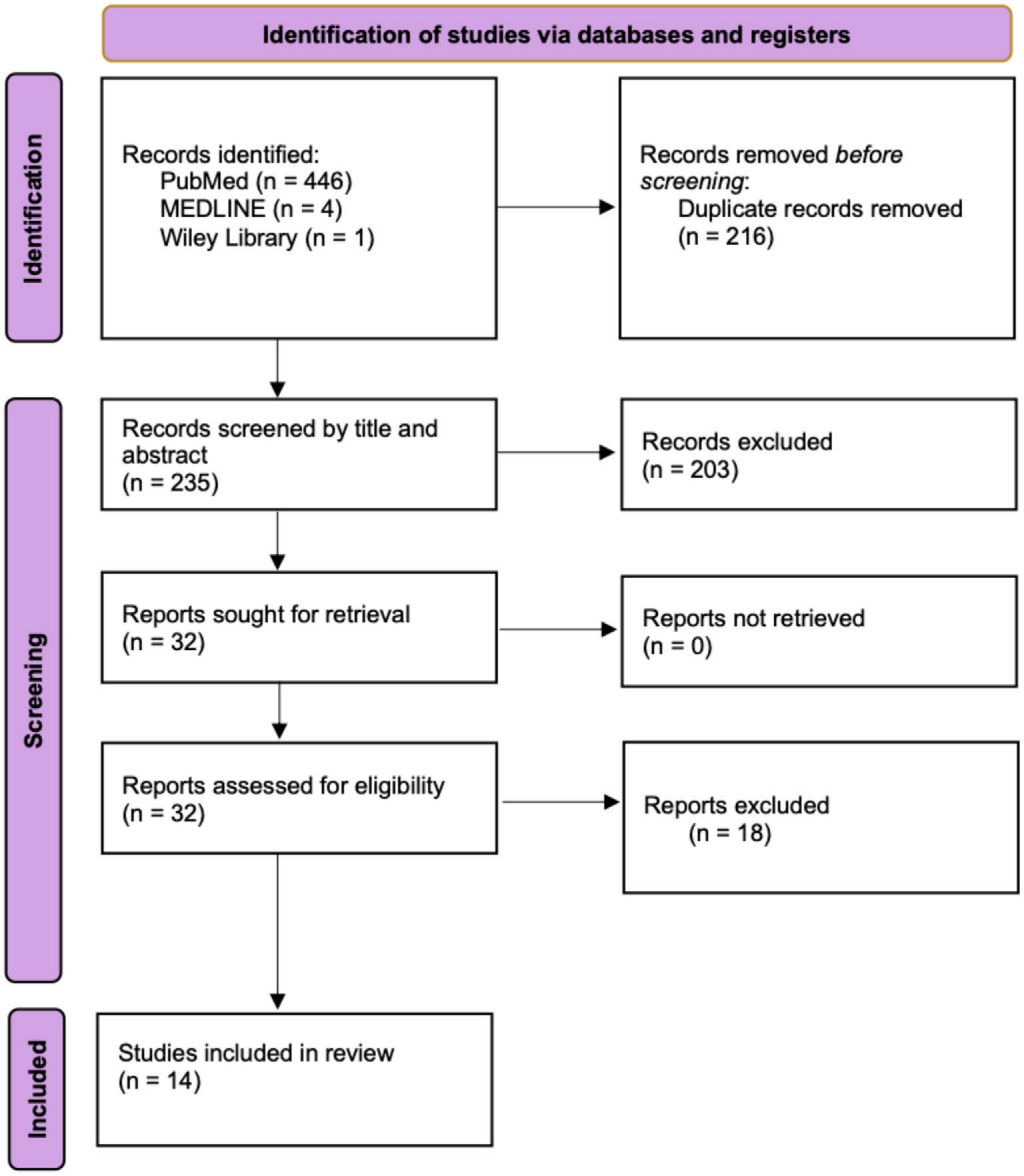
PRISMA diagram detailing the screening and selection process of the articles included in this review.

Inclusion criteria included full‐text studies published between 2015 and 2025 that investigated the outcomes of PRP or PRF injections within the periorbital region of healthy human adults for aesthetic purposes. Exclusion criteria included duplicates, review articles, nonhuman studies, articles published prior to 2015, studies not pertaining to the keywords contained in the search algorithm, studies that did not use purely injectable PRF or PRP on at least one side of the face, and studies outside the periorbital region (Figure 1).

### 2.3 Risk of Bias Assessment

Two independent reviewers evaluated the articles to ensure that they were pertinent to the study and adhered to the inclusion criteria. To screen for potential bias in the included papers, the Cochrane Risk of Bias‐2 tool was employed for randomized studies and the ROBINS‐I tool for non‐randomized studies. Both tools assess the quality of papers in terms of measurement tools, reported outcomes, completion of data, and deviation from the intended intervention.

## 3 Results

A total of 14 articles were included in this review (Table 1). The retrieved articles were analyzed for differences in the preparation process, injection technique and frequency, aesthetic and clinical improvement, efficacy parameters, patient satisfaction, and adverse events. An investigation into these characteristics will help to decipher the unique outcomes and benefits of each treatment modality, as well as highlight why certain studies displayed more or less favorable results.

**TABLE 1:** Characteristics of fourteen studies investigating the use of injectable PRP or PRF for aesthetic rejuvenation of the periorbital region.

Study design	Treatment	Methods	Measures	Outcome
Shashank and Bhushan [19] Case series	PRF	39 year‐old female Blood centrifuged at 800 rpm for 4 min 1.5 mL injected per side each session subdermally in the under‐eye region 25‐gauge blunt tip cannula using linear threading fanning technique 3 treatment sessions each 1 month apart Evaluation at the end of third session	Histological analysis to ensure presence of required cells VAS for aesthetic improvement in skin laxity, texture, pigmentation, and infraorbital volume	Visible filling of under‐eye hollows immediately postinjection, lasting about 2 weeks Patient reported reduced under‐eye skin laxity, pigmentation, and hollowing as well as improvement in skin texture No adverse effects reported
Mahmoodabadi et al. [20] Experimental clinical trial	PRF	8 men and women > 30 years‐old Blood centrifuged at 700 rpm for 5 min 3 cc total injected subdermally into periorbital region using a 27‐gauge cannula 1 treatment session 12‐week follow‐up	Wrinkle severity based on tissue volume and depth (Visioface 1000D) Patient satisfaction	Significant improvement in deep wrinkles; noticeable improvement of fine and small periorbital wrinkles, POH, and overall skin freshness Tissue volume size significantly decreased for both women and men on the right side (*p* = 0.027, *p* = 0.008, respectively) as well as on the left (*p* = 0.027, 0.010) All participants “eager” to recommend procedure afterwards Swelling in the injection site up to 1 day postinjection
Majewska [21] Retrospective study	PRF	10 women ages 32–45 Blood centrifuged at 60 g for 3 min 2 mL i‐PRF injected intradermally in periorbital region 4 mm 30‐gauge needles using mesotherapy technique 4 treatment sessions each 1 month apart 1 month follow‐up	Skin density (DUB SkinScanner) VAS for patient satisfaction with aesthetic improvement	Increased skin density in all patients following session 1 Increase in mean skin density for all groups: 1.66× higher compared to the baseline after the 2nd session, 5.08× higher after the 3rd session Average VAS score increased by 4.5 points (4–8.5) Mild bruising and slight pain at the site of injection, which diminished after 24 h
Atsu et al. [22] Prospective cohort study	PRF vs. PRP	55 subjects (52 women) ages 23–58 PRP (*n* = 23) and PRF (*n* = 32) groups assigned upon patient preference Blood centrifuged at 2000 rpm for 2 min PRP tubes contained 3.8% sodium citrate anticoagulant No mention of specific activation step for PRP 0.5 mL PRP or PRF injected intradermally using a 27–30 gauge needle 3 treatment sessions each 1 month apart Follow‐up at 1, 3, and 6 months	Self‐rated treatment efficacy VAS for pain measurement Skin topography: smoothness, roughness, scaliness, and wrinkles (Visioscan UVA light camera)	A significant superiority of PRF over PRP seen for canthal smoothness and wrinkles at 3 months (*p* = 0.025 and 0.028 respectively) which disappeared by 6 months No significant difference in canthal roughness and scaliness for PRP or PRF groups (*p* > 0.05) The groups did not significantly differ in self‐rated treatment efficacy (*p* = 0.743) or pain (*p* = 0.860) Redness (most common) bruising, burning, edema; the groups did not differ in the frequency of experience of adverse events
Majewska et al. [23] Single‐center, prospective, open‐label randomized study	I‐PRF vs. F‐PRF and C‐PRP vs. PRP LCC	20 subjects ages 30–60 Fitzpatrick skin types I–III Facial wrinkles Glogau class II or higher Randomly assigned treatment group Blood centrifuged at 55 g for 4 min 1 mL I‐PRF injected into the lower eyelid with 4 mm 30‐gauge needles using mesotherapy technique 3 treatment sessions with 4–6 weeks intervals in between Follow‐up at 1 month	Tissue, epidermal and dermal density, and thickness (DUB SkinScanner) Collagen and elastin content in dermis GAIS Patient satisfaction Likelihood to recommend treatment	Statistically significant increase in lower eyelid skin density (*p* = 0.0461) and thickness (*p* = 0.048317) following 3rd session As opposed to modalities tested in different facial regions, I‐PRF in the under eyes yielded the most substantial improvement skin condition Overall 60% of patients were “satisfied” and 40% “very satisfied” Redness, swelling, and bruising all subsided after 4 months
El‐Tahlawi et al. [24] Prospective cohort study	PRP vs. carboxytherapy	23 patients (22 female) ages 18–42 with periorbital dark circles (PODC) Fitzpatrick skin types III and IV First spin: 377 g for 10 min Second spin: 2504 g for 10 min 3.2% sodium citrate anticoagulant Activated with 0.1 mL calcium chloride per 1 mL plasma Injected using 32‐gauge needle into right periorbital papillary dermis (1.5–2.0 mm deep) at < 15° with mesotherapy technique 0.2 mL in the upper lid, 0.5 mL in the lower 4 treatment sessions in 1 week intervals 3 month follow‐up	Mean area percent of melanin via histopathological evaluation Longevity of results Pain scale Clinical improvement (photographic assessment by blinded physicians) PODC grade Patient satisfaction	PRP showed a significantly better response (*p* = 0.002), shorter downtime, and more tolerable side effects 46.6% reduction in area percentage of melanin, statistically significant better reduction of melanin compared to other group (*p* = 0.002) 26% displayed visible improvement in periorbital wrinkles Statistically significant satisfaction among physicians and patients (*p* = 0.001) Moderate pain at injection site and transient ecchymoses
Budania et al. [25] Randomized, prospective comparative study	Novel (single‐spin) vs. traditional (double‐spin) PRP	21 female patients ages 18–50 with bilateral POH Randomly divided into Group A “Novel” and B “Traditional” “Novel” centrifuged at 100 g for 10 min and activated by storing at 4°C for 15–30 min prior to injection “Traditional” centrifuged at 160 g for 10 min and again at 400 g for 10 min, activated in a 1:9 ratio of calcium gluconate:PRP 3.2% sodium citrate anticoagulant used for both 1–1.5 mL PRP administered at 0.05 mL/cm^2^ with 30‐gauge insulin syringe intradermal to subcutaneously in loose periorbital skin 3 treatments given in 4 week intervals Follow‐up 1 month after last session	Presence of VEGF, endostatin and platelet count Clinical improvement of POH (blinded physician assessment) VAS for POH severity Dermatology life quality index (DLQI)	Mean improvement in novel PRP was 52.33 ± 6.468 while that of conventional PRP was 53.14 ± 6.998; no significant difference between the two groups (*p* = 0.151) 86% of all participants displayed “good” (50%–74%) improvement; 14% showed “fair” (25%–49%) improvement; neither group had improvement > 75% or < 24% Novel and conventional VAS and DLQI scores significantly decreased from baseline to week 12 (*p* < 0.001 and *p* = 0, respectively) No significant difference in platelet count and VEGF obtained from either PRP; endostatin levels unmeasurable Mild pain and bruising, pain subsided within 1–2 h
Diab et al. [26] Comparative clinical study	PRP vs. PPP biofillers	40 female patients ages 21–40 with periorbital wrinkles and/or dark circles Fitzpatrick skin types II–III Citrate dextrose anticoagulant No specific mention of PRP activation First spin: 320 g for 15 min at 4°C Second spin: 1000 g for 5 min PRP injected intradermally into right periorbital region using 26‐gauge needles 2 treatment sessions 4 weeks apart Follow‐up 2 weeks after each session and 12 weeks after last session	GAIS Patient satisfaction (Likert scale) Glogau wrinkle scale for dark circle/wrinkle severity Wrinkle depth, roughness index, average melanin concentration	Significant improvement in periorbital wrinkles 2 weeks after the 2nd session, with significantly better results on plasma gel side (*p* = 0.009); no improvement in POH 2 weeks after the 2nd session, 20% of PRP patients were still unsatisfied, 17.5% were moderately satisfied and 42.5% were partially satisfied; no patients reported complete satisfaction Degree of improvement could not be maintained by 12‐week follow‐up Transient pain, erythema, ecchymoses, swelling, feeling of pressure; all resolved before next follow‐up
Badran et al. [27] Clinical trial	PRP	30 patients (25 female) ages 18–47 with POH and Fitzpatrick skin types III and IV First spin: 160 g for 10 min Second spin: 400 g for 10 min 1.5 mL trisodium citrate anticoagulant No activation step 1.5 mL PRP injected via 4–5 unit microinjections with 4 mm insulin syringes in the periorbital region, both superficially and intradermally 3 sessions in monthly intervals Follow‐up 1 month after 3rd session	POH severity grading Clinical POH improvement (blinded physician assessment) Patient satisfaction	Significant improvement in POH severity (*p* < 0.001); Grade II POH was the most substantial No patients displayed excellent improvement; 43.3% showed moderate or significant improvement and 56.7% showed no or slight improvement 63.3% of patients reported treatment satisfaction; 20% pleased; 16.7% neutral Temporary erythema, swelling, and ecchymoses
Iranmanesh et al. [28] Split‐face, randomized clinical trial	PRP vs. tranexamic acid with vitamin C	18 patients (16 female) with a history of POH recruited via simple random method First spin: 1800 g for 6 min Second spin: 2500 g for 15 min No mention of activation step 1 mL PRP injected in the infraorbital region intradermally on one side of face using 30‐gauge insulin syringes 3 treatments given in 3‐week intervals Follow‐up 3 months after last session	PGA for POH improvement Patient satisfaction measured via VAS	Average VAS for PRP was 4.83; not significantly higher than that of vitamin C (*p* = 0.58) 44.4% of PRP patients (*n* = 8) displayed moderate improvement (51%–75%), while 22.2% of PRP patients (*n* = 4) reported excellent (76%–100%) improvement No severe complications reported
Sadiq et al. [29] Randomized control trial	PRP vs. PPP biofillers	42 patients ages 18–65 with Fitzpatrick skin types I–V and POH Randomly assigned PPP (*n* = 21) or PRP treatment (*n* = 21); 7 males and 14 females in each group First spin: 3500 rpm for 15 min Second spin: 1500 rpm for 5 min No mention of activation step 1 mL acid citrate dextrose anticoagulant 2 mL PRP injected around each eye using insulin syringes 2 treatment sessions with 2‐week intervals Follow‐up 2 weeks posttreatment	Photometric pigmentation scale Clinical improvement of dark circles (blind physician assessment) Patient satisfaction	PPP patients displayed greater clinical improvement, satisfaction, and fewer complications 57% of PRP patients were fairly satisfied, 28.6% moderately satisfied, and only 4.76% completely satisfied (*p* = 0.001) 33.3% of PRP patients displayed fair clinical improvement, while 57% had good clinical improvement PRP side effects included pain and bruising
Ozer and Colak [30] Experimental clinical study	PRP	9 female patients with complaints of infraorbital darkness Blood centrifuged at 1630 g for 5 min Blood: anticoagulant (citrate dextrose) ratio of 9:1 No activation step Mean of 4.8 mL PRP injected infraorbitally each session 3 treatment sessions in 1‐month intervals Followed up over 9 month period	Patient‐reported satisfaction regarding facial appearance, skin quality, psychological and social function, aging appearance appraisal, and overall satisfaction with outcome	FACE‐Q satisfaction and quality of life modules displayed statistically significant improvement in all modules following PRP injections, including: overall facial appearance (*p* < 0.001), satisfaction with skin quality increased (*p* < 0.05), aging appearance appraisal (*p* < 0.05), and social/psychological function (*p* < 0.05) Mean outcome satisfaction was 83.33 ± 16.25 (out of 100) Transient ecchymosis and edema which improved during follow‐up
Neinaa et al. [31] Retrospective clinical comparative study	PRP vs. PPP biofillers	68 females with dark circles and/or tear trough deformity First spin: 3000 rpm for 15 min Second spin: 1500 rpm for 5 min 1:10 sodium citrate anticoagulant PRP activated with 10% calcium gluconate (0.01 mL per 1 mL PRP) 1 mL PRP injected intradermally in left infraorbital region using insulin syringes 3 treatments in 2‐week intervals Monthly follow‐ups for 3 months	Assessment of platelet concentration Degree of infraorbital hyperpigmentation Skin texture and homogeneity TTRS Global assessment for degree of improvement Patient satisfaction	Both groups displayed a significant decrease in mean degree of hyperpigmentation and TTRS (*p* < 0.001), PPP significantly more reduced (*p* < 0.001) All PRP patients reported some degree of improvement (slightly‐greatly) in texture and homogeneity 85.29% of PRP patients reported some degree of satisfaction (slightly‐very) Tolerable pain at injection site, temporary edema, and transient ecchymosis
Banihashemi et al. [32] Clinical trial	PRP	23 female patients with Glogau skin score of II to IV aged 35–55 First spin: 2000 g for 2 min Second spin: 4000 g for 8 min 5 mL plasma activated with 0.5 mL 10% calcium gluconate 1 cc PRP injected both subdermally and intradermally into the periorbital area and crow's feet 2 treatment sessions with 3‐month intervals 3 and 6 month follow‐ups	Skin wrinkles, darkness, moisture, pores, spots, and elasticity Patient satisfaction	Moderate‐excellent improvement in dark circles: 47.8% of patients at 3 months and 60.9% at 6 months Moderate‐excellent improvement in wrinkles: 73.9% of patients at 3 months and 78.3% at 6 months Physician evaluations at both follow‐ups showed significant improvement in dark circles (*p* = 0.008, *p* = 0.025) Skin scans displayed significant improvement in wrinkles from baseline to 6‐months (*p* = 0.007) Edema, bruising, and fainting (1 patient)

*Note:* Studies published between 2015 and 2025 meeting inclusion and exclusion were reviewed. Five studies evaluated the effects of PRF and ten evaluated PRP, as one study evaluated both.

Abbreviations: C‐PRP, Concentrated PRP; F‐PRF, Fluid‐PRF; GAIS, Global Aesthetic Improvement Scale; I‐PRF, Injectable PRF; PGA, Physician Global Assessment; POH, periorbital hyperpigmentation; PPP, platelet‐poor plasma; PRP LCC, low‐centrifugation concept PRP; TTRS, Tear Trough Rating Scale; VAS, Visual Analogue Scale.

### 3.1 Preparation and Protocol

Multiple centrifugation protocols were identified in the literature, varying in speed, time, temperature and centrifugation rounds. Centrifugation speed was measured in either revolutions per minute (rpm) or relative centrifugal force/G‐force (g). As for studies involving PRF, single‐spin methods were employed and varied between 700–2000 rpm and 55–60 g for 2–5 min [19, 20, 21, 22, 23]. Mahmoodabadi et al. centrifuged PRF for the longest period but lowest speed, at 700 rpm for 5 min, followed by Shashank and Bhushan at 800 rpm for 4 min, and Atsu et al. at 2000 rpm for 2 min [19, 20, 22]. Majewska and Majewska et al. centrifuged the samples at 60 g for 3 min and 55 g for 4 min, respectively [21, 23]. All studies prepared and utilized injectable PRF (i‐PRF), as a liquid formulation is necessary in order to be injected. Majewska et al. did compare i‐PRF with fluid‐PRF (F‐PRF), which underwent a second centrifugation; however this review did not focus on those findings as the F‐PRF was only injected into the cheeks [23].

In PRP studies, the double‐spin method was most often utilized, with first‐round speeds ranging from 3000–3500 rpm and 55–2000 g for 2–15 min and second‐round spins from 400–4000 g and 1500 rpm for 4–15 min [24, 26, 27, 28, 29, 30, 31, 32]. Conversely, Atsu et al. and Ozer and Colak utilized single‐spin methods to prepare PRP, as blood was centrifuged once at 2000 rpm for 2 min and 1630 g for 5 min, respectively [22, 30]. Budania et al. compared two distinct PRP preparations, the “novel” formulation which was centrifuged once at 100 g for 10 min, and the “traditional” formulation which was centrifuged at 160 g for 10 min followed by 400 g for 10 min [25].

Some studies centrifuged or stored PRP at a lower temperature (4°C) to improve quality and potentially slow its metabolism, but it did not enhance its efficacy; Budania et al. reported an insignificant difference between the platelet count and VEGF obtained from the temperature‐activated PRP versus the traditional PRP [25, 26].

Most other protocols state that PRP was activated with either calcium chloride or gluconate. El‐Tahlawi et al. used a 1:10 ratio of calcium chloride to plasma, while Budania et al., Neinaa et al., and Banihashemi et al. opted for 10% calcium gluconate in similar ratios [24, 25, 31, 32]. The remaining studies either excluded or did not explicitly mention an activation step [22, 26, 27, 28, 29, 30]. It should be noted that this heterogeneity in centrifugation protocols limits this review in terms of direct comparison between studies.

### 3.2 Injection Technique

Studies also varied in the amount of product injected as well as the techniques used. Both Shashank and Bhushan and Mahmoodabadi et al. injected 3 mL PRF per treatment session using blunt‐tip cannulas. However, Shashank and Bhushan completed three treatments in 1 month intervals, injecting a total of 9 mL PRF, whereas Mahmoodabadi et al. only completed one treatment [19, 20]. Other studies instead used 4 mm 27–30‐gauge insulin syringes and injected between 0.5 and 2 mL per session, with varying total amounts [21, 22, 23]. Majewska injected a total of 8 mL over four treatment sessions in monthly intervals, while Atsu et al. and Majewska et al. injected a total of 1.5 and 3 mL, respectively, in three sessions with monthly intervals [22, 23].

As for injection depth, Atsu et al. and Majewska injected intradermally, while Shashank and Bhushan and Mahmoodabadi et al. injected subdermally [19, 20, 21, 22]. Majewska et al. did not specify injection depth for PRF [23].

All PRP studies used 26–32‐gauge insulin syringe needles for delivery, but varied in injection amount and frequency. Ozer and Colak administered a mean of 4.8 mL per session across three treatments in monthly intervals [30]. Budania et al. and Badran et al. each injected approximately 1.5 mL per session, also completing three monthly treatments [25, 27]. Sadiq et al. delivered 2 mL over two sessions separated by 2 weeks, totaling 4 mL [29]. Neinaa et al. and Iranmanesh et al. used slightly lower doses, 1 mL/session, and completed three treatments in 2–3 weeks intervals [28, 31]. Banihashemi et al. also injected 1 mL periorbitally, including the crow's feet, but only performed two sessions separated by 3 months [32]. El‐Tahlawi et al. administered 0.7 mL per session over four weekly sessions, totaling 2.8 mL [24].

PRP was injected intradermally in all studies except those by Sadiq et al. and Ozer and Colak, which did not specify injection depth. Badran et al. additionally reported superficial injections, while Banihashemi et al. combined intradermal with subdermal injections [27, 32].

### 3.3 Aesthetic and Clinical Improvement

All five PRF studies reported some degree of periorbital aesthetic improvement. However, the use of distinct amounts, treatment numbers, and injection techniques makes it difficult to determine an ideal protocol to yield significant results. The amount injected seemingly did not affect aesthetic results or patient satisfaction: while Majewska and Shashank and Bhushan injected a total of 8–9 mL, Atsu et al., Mahmoodabadi et al., and Majewska et al. all injected a total of 3 mL or less, yet all studies displayed noticeable improvement in skin quality. Overall, two studies found significant improvement in under‐eye wrinkles, two found significant increases in under‐eye skin density, and only one reported reduced pigmentation and immediate improvement of under‐eye hollows, which was likely due to transient swelling [19, 20, 21, 22, 23]. Mahmoodabadi et al. observed a statistically significant reduction in wrinkle depth and tissue volume on both sides of the face (*p* < 0.05), supported by Visioface 1000D analysis [20]. Similarly, Majewska et al. demonstrated a statistically significant increase in lower eyelid skin density (*p* = 0.0461) and thickness (*p* = 0.048317) following the third treatment using DUB SkinScanner imaging [23]. With the same imaging tool, another study by Majewska achieved a 5.08‐fold increase in mean skin density after three sessions, and an average VAS score of 8.5 [21]. Atsu et al. found a significant superiority of PRF over PRP for canthal smoothness and wrinkle reduction at the 3‐month mark (*p* = 0.025 and *p* = 0.028, respectively), but no difference in canthal roughness and scaliness (*p* > 0.05).

Ten studies investigated PRP, nine of which assessed POH. Quantitative measures consistently supported a reduction in melanin or pigmentation severity [24, 27, 30, 31, 32]. El‐Tahlawi et al. reported a 46.6% mean reduction in melanin area percentage (*p* = 0.002) following four sessions, but wrinkle improvement in only 26% of patients [24]. At 6 months, Banihashemi et al. observed moderate‐to‐excellent improvement in dark circles in 60.9% of patients and wrinkle reduction in 78.3% (*p* = 0.007) [32]. Badran et al. also found significant POH improvement (*p* < 0.001), with 43.3% showing moderate‐to‐significant change [27]. Conversely, Diab et al. documented no significant pigmentation improvement but a notable reduction in wrinkle indentation measured by 3D Antera from the first to the second visit (*p* = 0.037) and according to the GAIS scale 2 weeks after the second session (*p* < 0.05) [26]. Iranmanesh et al. observed 44.4% of patients with moderate (51%–75%) improvement and 22.2% with excellent (76%–100%) improvement via physician assessment [28]. Ozer and Colak also stated significant improvement in skin quality; however, this is a more generalized parameter that may not be specific to wrinkles or pigmentation [30].

### 3.4 Modes of Measurement

The degree of aesthetic improvement was not measured through uniform, objective means across all studies. For PRF, studies by Majewska and Majewska et al. utilized an ultrasound system (DUB Skin Scanner) to quantify tissue density and thickness before and after treatment, which further substantiated the subjective results (i.e., visible improvement, patient satisfaction). Similarly, Atsu et al. utilized topographical measurements to quantify skin texture parameters (Visioscan VC 20plus), and Mahmoodabadi et al. used a skin analysis software (Visioface 1000D) to classify wrinkle severity based on tissue volume and depth. Only Shashank and Bhushan relied solely on subjective measures (VAS scores) to determine efficacy.

For PRP, only two studies employed objective modes of aesthetic measurements: El‐Tahlawi et al. evaluated area percentage of melanin via histopathology whereas Diab et al. used an Antera 3D camera to quantify wrinkle depth, skin roughness and average melanin concentration [24, 26]. All other PRP studies utilized subjective parameters, like patient satisfaction, clinical evaluation, and aesthetic grading scales.

### 3.5 Patient Satisfaction

Both treatment modalities generally yielded increased patient satisfaction compared to baseline. While every PRF study reported patient satisfaction, some reports were more anecdotal than others [20]. Majewska and Majewska et al. both recorded substantial increases in patient satisfaction using aesthetic scales such as the VAS and GAIS, respectively [21, 23]. It should be noted, however, that the average GAIS score of 2.75 (“very much improved”) reported by Majewska et al. reflects a composite of PRF treatments performed, not limited to the under eyes.

PRP studies, however, varied more in regard to patient satisfaction. Budania et al. demonstrated significant patient satisfaction at week 12 via VAS and DLQI scores (*p* < 0.001 and *p* = 0, respectively) [25]. Additionally, El‐Tahlawi et al. exhibited statistically significant satisfaction among both physicians and patients (*p* = 0.001) [24]. Ozer and Colak utilized FACE‐Q satisfaction and quality of life modules to display statistically significant patient satisfaction in all respects [30].

Several other PRP studies reported relatively high degrees of patient satisfaction, despite some variance [27, 29, 32]. Although more than half of the patients in Iranmanesh et al. reported some degree of improvement posttreatment, the average VAS score (4.83) was not significantly higher than that of the vitamin C group [28]. According to the study by Diab et al., none of the 40 patients displayed complete satisfaction with PRP injections [26]. Overall, there is minimal evidence suggesting a stronger patient preference for either; the only study that directly compared PRP and PRF injections in the periorbital region found no significant difference in patient‐rated efficacy scores (*p* = 0.743) or VAS scores (*p* = 0.860) [22].

### 3.6 Adverse Event Profile

Adverse events resulting from both treatment modalities were transient and benign, mostly limited to swelling, redness, and bruising. Only one study reported a patient fainting during PRP treatment [32]. Atsu et al. reported that PRF and PRP patients did not differ in terms of adverse event frequency, nature, or pain (*p* = 0.860) [22]. All studies reported resolution of adverse events within the treatment timeline.

## 4 Discussion

To our knowledge, this is the first systematic review to compare the efficacy of both PRP and PRF in treating the periorbital region for aesthetic purposes. Despite yielding some promising results, the evidence supporting the use of both modalities for periorbital rejuvenation is limited and somewhat inconsistent. Although there is more substantial data surrounding PRP, it remains slightly underwhelming. This underscores the need for further discussion regarding how to optimize their use and which treatment should be preferred.

Given the aesthetic outcomes, PRF may serve as a potentially useful adjunct for ameliorating skin texture concerns, such as wrinkles or crepiness. As for PRP, there is far more data supporting its use to mitigate hyperpigmentation, rather than PRF. Yet, this is likely due to the novelty of PRF and the lack of adequate studies addressing POH. Overall, both treatments generally displayed little to no adverse effects and considerable patient satisfaction, regardless of the amount used, depth, or protocol. Thus, any hypothetical superiority of PRF over PRP would depend on both the degree and longevity of results.

Although increased longevity was one of the very reasons for its development, it is unknown whether PRF actually accomplishes this goal due to the lack of long‐term observation. Atsu et al. provided the most long‐term PRF follow‐up at 6 months, where it was found that the initial significant improvement in canthal smoothness and wrinkles had disappeared [22]. Conversely, Banihashemi et al. still found significant improvement of dark circles (*p* = 0.025) and wrinkles (*p* = 0.007) 6 months post‐PRP treatment [32]. Other PRF studies that reported sustained improvement had much short‐term follow‐ups, between 1 and 3 months at most [20, 21].

These discrepancies challenge the proposed longevity of PRF and whether patients are satisfied with the procedure or just the transient appearance of swelling. A study by Liang et al. demonstrated lasting patient satisfaction and improvement of facial skin texture (> 1 year) from combined nanofat‐PRF injections. However, this likely speaks more to the effects of nanofat‐derived stem cells which the PRF had merely enhanced [33]. In fact, both PRP and PRF have demonstrated more impressive aesthetic results and patient preference when used as adjuncts in combined treatments, such as microneedling, lasers, or HA filler [12, 34, 35]. Various other studies investigating pure PRF for facial skin rejuvenation have reported lasting results up to the 3‐month mark, but no later [16, 36].

The lack of long‐term data also calls into question how quickly PRF concentrate is resorbed once injected, and whether certain protocols are more effective at prolonging release. Although its fibrin mesh provides advantageous growth factor delivery in comparison to PRP, studies have shown that PRF typically gets resorbed by the body within 2 weeks [37, 38]. Some studies have demonstrated that these resorption properties may be extended for up to 6 months by heating the PPP layer during preparation, thus overcoming the rapid degeneration of platelet concentrates in the body [37]. When the same heating process is undergone during PRP preparation, 50% of the resulting matrix is still resorbed within a week [39]. Although this method has not been investigated for use in the periorbital region, it showcases PRF's potential to yield significant longevity, possibly making it a more desirable option. Moreover, histological studies have shown uneven cell type distribution within PRF fractions, which may also affect longevity, degree of improvement, and ultimately explain result discrepancies [40]. Thus, future studies should analyze the histological composition prior to injection to ensure accurate comparison between patients.

A critical limitation to this study is the lack of uniformity among centrifugation protocols and aesthetic improvement parameters. The varying centrifuge speeds, times, and tube characteristics complicate the ability to directly compare studies or consistently reproduce outcomes. These inconsistencies may also account for some of the variability observed in reported efficacy. This highlights the need for more studies that employ standardized preparation and reporting guidelines, as well as objective measurements, such as surface metrology [41]. Quantifying aesthetic results in this manner would allow for a more accurate comparison of treatment modalities.

In conclusion, PRF demonstrates favorable outcomes in improving periorbital skin texture while avoiding the potential side effects associated with other common in‐office procedures. Yet, it lacks the depth of study that PRP has accumulated throughout the years and does not pose a significantly superior aesthetic benefit thus far in the literature. Ultimately, injectable PRF will require more randomized controlled trials using objective modes of measurement in order to better assess its efficacy. Despite being less effective, both treatments remain suitable options for patients who are hesitant to undergo HA injections due to concerns about allergies or adverse effects, as well as patients who desire a more modest, natural approach to facial rejuvenation without dramatic results.

## Ethics Statement

The authors confirm adherence to the journal's ethical policies as stated on the journal's author guidelines page. No ethical approval was required for this review article as there was no original research conducted.

## Consent

The authors have nothing to report.

## Conflicts of Interest

The authors declare no conflicts of interest.

## Data Availability

The authors have nothing to report.
